# Targeting protein function: the expanding toolkit for conditional disruption

**DOI:** 10.1042/BCJ20160240

**Published:** 2016-08-30

**Authors:** Amy E. Campbell, Daimark Bennett

**Affiliations:** * Department of Biochemistry, Institute of Integrative Biology, University of Liverpool, Crown Street, Liverpool L69 7ZB, U.K.

**Keywords:** biochemical techniques and resources, cellular targeting, chemical biology, protein dynamics, optogenetics

## Abstract

A major objective in biological research is to understand spatial and temporal requirements for any given gene, especially in dynamic processes acting over short periods, such as catalytically driven reactions, subcellular transport, cell division, cell rearrangement and cell migration. The interrogation of such processes requires the use of rapid and flexible methods of interfering with gene function. However, many of the most widely used interventional approaches, such as RNAi or CRISPR (clustered regularly interspaced short palindromic repeats)-Cas9 (CRISPR-associated 9), operate at the level of the gene or its transcripts, meaning that the effects of gene perturbation are exhibited over longer time frames than the process under investigation. There has been much activity over the last few years to address this fundamental problem. In the present review, we describe recent advances in disruption technologies acting at the level of the expressed protein, involving inducible methods of protein cleavage, (in)activation, protein sequestration or degradation. Drawing on examples from model organisms we illustrate the utility of fast-acting techniques and discuss how different components of the molecular toolkit can be employed to dissect previously intractable biochemical processes and cellular behaviours.

## INTRODUCTION

Genetic manipulation, which operates at the level of the gene or its transcribed product, has proven to be indispensable for the identification of molecular components required for biological processes and understanding how these components act together to construct functional cells, tissues and organisms. There are now a myriad of tools for mutational analysis that have been accumulated for over a century, fuelling gene discovery through forward genetic screens and facilitating reverse genetics to probe gene function [1–3]. Recent developments, in particular CRISPR (clustered regularly interspaced short palindromic repeats)-based approaches and RNAi, promise to further transform our understanding by facilitating high-throughput reverse genetics and gene editing with nucleotide-level precision [4,5]. Gene overexpression technologies have also become extremely advanced, featuring a high level of spatial and temporal control that has enabled a range of developmentally targeted gain-of-function studies. Heterologous gene expression systems, such as yeast Gal4/UAS (GAL4 upstream activating sequence), have proven to be particularly versatile in this regard [6]. Yet, quite often, the genetic tools used to perturb gene function are not able to keep pace with dynamic biological events that can act over different timescales from less than a second to many hours or days, depending on the process.

Processes acting over short times are particularly recalcitrant to genetic analysis because there is a considerable delay between perturbations at the transcriptional or post-transcriptional level and the corresponding effect on the encoded protein. Consequently, genetic approaches are typically incapable of selectively disrupting the encoded protein of interest at the time when the effects of experimental manipulation are measured. This is particularly a problem for analysing the function of proteins required at multiple points of the same process. A partial solution to this problem might be to monitor the process under investigation in real-time, so as to capture more information about the biological effects than can be revealed at a fixed time point [7]. Yet significant limitations remain, especially for the analysis of protein function *in vivo*. For instance, mutation of a gene required for development might result in early lethality making later processes impossible to analyse. Alternatively in the case of transgenic RNAi, which typically results in partial loss-of-function, it may not be possible to drive expression of the transgene early enough for knockdown to occur before the process has already taken place. This is particularly a problem early in development, where it can take a considerable time for the maternal contribution of RNA and protein to be exhausted. Another major consideration is the existence of compensatory homoeostatic mechanisms that may circumvent the requirement for the protein under investigation. Although this is often a reason cited for lack of knockout phenotypes in mice, the underlying mechanisms are frequently not well described. Studies employing fast-acting methods have the potential to resolve such issues, as illustrated by a study of the cell-surface glycoprotein CD44 [8]. CD44 isoforms act as co-receptors for the receptor tyrosine kinases c-Met and VEGFR-2 (vascular endothelial growth factor receptor 2), but do not produce overt phenotypes when knocked out in mice [8]. Using blocking antibodies, it was shown that acute disruption of CD44v6 inhibited cell proliferation and c-Met activation in wild-type mice, but that ICAM-1 (intercellular adhesion molecule 1) compensated for the CD44v6 isoform in CD44-null mice [8]. This study illustrates that rapid blockade of protein function can be a powerful way of resolving problems associated with slow-acting or constitutive methods of gene disruption. However, only a minority of proteins are currently open to pharmacological manipulation and the development of specific blocking reagents for every protein of interest based on their intrinsic properties is a long way away.

A successful strategy that has been adopted by the research community over the last few years to increase the range of targets that can be manipulated pharmacologically has been to take well-characterized ligand-interaction domains from heterologous systems, and genetically engineer them into proteins of interest. In parallel to this chemical genetics approach, researchers have also found ways of incorporating domains responsive to other triggers, such as light, temperature and pH. This has spawned a new generation of tools that act directly at the level of the expressed protein and have the potential to provide insight into acute perturbations, give access to analysis over short times and allow reversible switching. Such tools can be broadly categorized according to their mode of action: those that disrupt protein activity through complex (de)formation, (in)activate proteins by induced splicing or cleavage, or directly target a protein for degradation through the endogenous cell machinery. In the present review, we focus primarily on tools for conditional control of protein function that fall into one of these three categories and are fast acting, providing examples of their application as a guide to researchers considering their use.

## COMPLEX FORMATION

**Figure 1:**
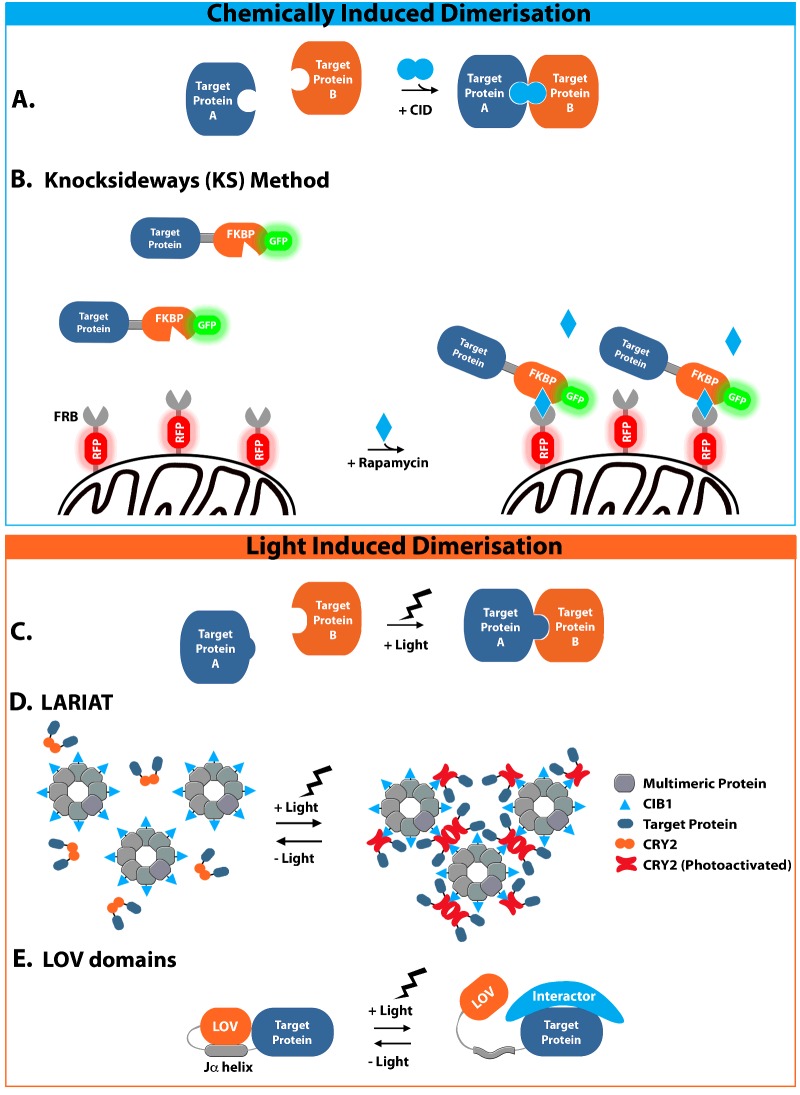
Schematic representation of methods for the control of protein activity by induced complex formation Methods described here can be broadly categorized by their method of induction. (**A**) Generalized mechanism for small-molecule-mediated approaches involving small-molecule CIDs to sequester protein activity by promoting (dis)aggregation, altering transcription or by changing the subcellular localization of target proteins. (**B**) A specific example of this approach is illustrated by the knocksideways (KS) method, which involves the rapid sequestration of target proteins to the mitochondria through rapamycin-mediated interaction between the FKBP-tagged target protein and the mitochondrially localized mito-RFP-FRB trap. (**C**) The generalized mechanism for light-based approaches induced by exposure to a specific wavelength of light. More specific examples of light-based methods are also illustrated in (**D**) and (**E**). (**D**) Schematic depiction of the LARIAT method, whereby target proteins tagged with CRY2 are sequestered into large protein complexes upon a light-induced conformational change to produce the photoactivated form of CRY2. This interacts with CIB1-bound multimeric complexes to aggregate the target protein and sequester activity. (**E**) Representation of LOV-domain-based approaches, which involve a light-induced conformational change leading to unravelling of the Jα helix and the loss of its interaction with the LOV domain leaving the target protein free to bind interactors and perform its function.

The promotion of interactions between proteins of interest represents a powerful strategy for the conditional control of protein activity. There are a number of different mechanisms by which such interactions can be engineered to spatiotemporally regulate protein activity in response to different stimuli, in a way that is both precise and fast acting (Figure 1, Table 1).

**Table 1:** Summary of methods for conditional control of protein complex formation

			Can be induced by			
Technique	Protein disruption	Timescale	Small molecule/hormone	Light	Temperature	pH
Chemically induced dimerization (CID)	Sequestration/(in)activation	min/h	✓ e.g. FK1012, rapamycin	✗	✗	✗
Knocksideways (KS)	Sequestration	min	✓ Rapamycin	✗	✗	✗
Light-activated dimerization	Sequestration/(In)activation	s/min	✗	✓	✗	✗
Light-activated reversible inhibition by assembled trap (LARIAT)	(In)activation	s	✗	✓ Blue light: 450–495 nm	✗	✗
Light-oxygen-voltage (LOV) domains	(In)activation	s/min	✗	✓ Blue light: 450–495 nm	✗	✗

### Chemically induced dimerization

One of the first mechanisms involving engineered protein complex formation utilized small molecules, referred to as chemical inducers of dimerization (CIDs), which simultaneously bind domains engineered into two proteins of interest, bringing them into close proximity and promoting their interaction (Figure 1A, Table 1). Methods involving CIDs can influence protein activity by promoting (dis)aggregation, altering transcription or by changing the sub cellular localization of target proteins. The application of CIDs to control protein activity and study protein function spans the last two decades, with the majority of applications utilizing naturally occurring CIDs, found to dimerize specific protein–ligand pairs [9–11]. For a small molecule to be successful in CID approaches it must have the ability to simultaneously bind two proteins, and therefore must have two high-affinity highly specific protein-binding domains, joined in a way that allows both target proteins to bind and interact [12].

#### FK1012

The first, naturally occurring CID, FK1012, was reported in 1993 [14]. FK1012 is a derivative of the immunosuppressant drug FK506, which was found in 1991 to be capable of binding calcineurin and FK506-binding protein [12] (FKBP12) with high affinity [13]. FK1012 is a synthetic dimer of FK506 that lacks the intrinsic biological activity of FK506 and has since been utilized as a CID to bind multiple FKBP12 domains [14], bringing target proteins together in a defined reversible fashion and demonstrating the subtleties required for successful CID design [12]. The initial CID concept was demonstrated through a system in which addition of FK1012 activated the endogenous T-cell signalling cascade, via fusion of FKBP12 to the proximity-regulated ζ-chain of the T-cell receptor, leading to receptor aggregation and subsequent activation [14]. FK1012 and other CIDs capable of dimerizing a single protein domain, discovered in the years following, have since been applied to the study of many important cellular processes to, for example, induce apoptosis via aggregation of the Fas membrane signalling protein [15] or regulate transcription through ligand-dependent (dis)association of transcriptional activators with promoter regions [16].

#### Rapamycin

Although the first CIDs were only capable of homodimerization, these approaches could, in theory, be used to generate heterodimers if two proteins of interest were tagged with the same domain. The result would, however, be a mixture of heterodimers and homodimers of the two individual proteins of interest. The development of methods involving naturally occurring heterodimerizers therefore followed, with the most notable heterodimerizer rapamycin dominating the field since its discovery [17]. Rapamycin is an immunosuppressant drug that selectively binds both FKBP12 and FKBP–rapamycin) associated protein (FRAP)/mammalian target of rapamycin (mTOR) [18]. The FKBP and FKBP–rapamycin-binding (FRB) domains of these proteins, respectively, are sufficient for binding and retain the binding affinity of the full-length proteins [19]. A key step in the application of rapamycin as a CID was the production of rapamycin derivatives, known as ‘rapalogs’, which have a much lower affinity for endogenous proteins, thereby circumventing rapamycin's immunosuppressive activity. In parallel, the rapamycin-binding regions from FKBP12 and FRAP/mTOR were remodelled to bind the rapalog at nanomolar affinity, providing an orthologous rapamycin system for CID applications [20–22]. These rapalogs have since been used to conditionally dimerize proteins to interrogate many different biological processes. Notable examples include the study of mitosis, in which rapamycin-induced binding of the endoplasmic reticulum and Golgi membranes showed that these structures remain segregated during mitosis in mammalian cells [23], and also the study of phosphoinositides and their roles in endocytosis and intracellular trafficking [24,25].

One specific application of rapamycin-mediated control in mammalian cells is the knocksideways (KS) method (Table 1), which acutely sequesters protein activity through a change in subcellular localization. The KS method is capable of rapidly re-routing target proteins containing an FKBP domain to the mitochondria on a timescale of seconds, through rapamycin-induced binding to an FRB-containing protein with a mitochondrial targeting signal (mito-FFP-FRB) (Figure 1B) [26]. This method utilizes the principle of mitochondrial re-routing, whereby the protein of interest accumulates on the outer mitochondrial membrane in a way that, providing the new localization is not compatible with protein function, sequesters protein activity, but remains tolerable to cells [27]. In an initial proof-of-principle study, the KS method was used to study the role of two subunits of the adaptor protein (AP) complexes of clathrin-coated vesicles AP-1 and AP-2 [26]. Robinson et al. [26] used rapamycin-induced re-routing of AP-1 and AP-2 to the mitochondria, in combination with siRNA knockdown of the endogenous protein, to demonstrate the requirement for both proteins in the endocytosis pathway. Although the phenotype of AP-2 sequestration was similar to that resulting from siRNA approaches alone, the corresponding phenotype observed for AP-1 was distinct from that of the siRNA knockdown and is accredited to more rapid depletion achieved in the KS approach [26]. The effectiveness of the KS approach for rapid changes in protein activity has since been demonstrated in a number of varied applications to fast-acting processes in mammalian cells. For example, Cheeseman et al. [28] used the rapamycin-mediated approach to specifically remove TACC3-ch-TOG-clathrin complexes from the mitotic spindle within a timescale of 5 min following rapamycin addition. By re-routing these complexes to the mitochondria and away from mitotic spindles at defined stages in mitosis they were able to deduce their role in maintaining tension in kinetochore fibres, which are essential for correct segregation of chromosomes. Again, this phenotype was distinct from that observed with siRNA alone, demonstrating the utility of the KS approach.

CIDs offer an efficient way to control the dynamics of processes reliant on oligomerization. However, the same principles can also be used for the opposite mode of control, in which processes are inhibited by oligomerization and activated upon addition of a ligand that dissociates the complexes. To enable this mode of control, Rollins and colleagues identified an FKBP12 mutant F36M-FKBP (F_M_) with the ability to form discrete dimers that can be dissociated rapidly upon addition of ligand [22]. Using these tools, Al-Bassam et al. [29] were able to develop a novel pulse–chase system in which exogenous F_M_- tagged membrane proteins were accumulated gradually in the endoplasmic reticulum, and sequestered by the formation of aggregates. Within minutes of small-molecule ligand addition, the F_M_ domains dissociated and the accumulated membrane proteins could be simultaneously released for synchronous continuation along the secretory pathway [29]. Through this method, Al-Bassam et al. [29] were able to study proteins in a specific phase of the secretory pathway without interference from proteins in other phases of the pathway and thus overcome a major problem associated with studying this dynamic process.

In order for CID to be successful, target proteins must be considered on a case-by-case basis and prior knowledge of protein function is usually required in order to achieve thorough inactivation. With nanomolar affinities between ligand–protein pairs, CID approaches have high specificity and high efficiency. However, this puts them at a disadvantage in terms of reversibility, as they often require an additional ligand that competes for binding to relieve protein inactivation/sequestration. Effects are often irreversible [30]. Although CID approaches were initially developed and demonstrated *in vitro*, these applications have since been developed to allow *in vivo* studies [31]. However, the requirement for exogenous small ligand addition and resulting potential for off-target effects somewhat limit the practicality of such applications. Also, although the KS approach demonstrates the ability for CID approaches to operate on a timescale of minutes, generally CID based methods range from minutes to hours, limited by the requirement for efficient uptake of the chemical inducer, and are consequently not among the fastest acting tools for temporal control of protein dynamics.

### Light-induced dimerization (LID)

Another way in which protein dimerization can be induced is via light-based methods, which take advantage of naturally occurring photosensitive protein domains that dimerize upon exposure to a certain wavelength of light (Figure 1C, Table 1). Although maintaining the flexibility of CID approaches in terms of the response elicited and the many ways in which protein function can be disrupted, light-based methods generally overcome many of the limitations of CID. In particular, they provide improved spatiotemporal precision, mitigate the requirement for exogenous small molecule addition and operate on a timescale of seconds. Genetically encoded light-based (optogenetic) approaches have vastly expanded within the last decade from just a few applications to a whole toolbox of techniques with which to control protein activity [32]. Like CID, the first methods involving light-induced dimerization (LID) took advantage of naturally occurring photosensitive proteins, often discovered initially in plants, known as phytochromes and cryptochromes.

#### Phytochromes

Phytochromes are photoreceptive pigments encoded by small multigene families in plants and bacteria where they monitor red/far-red wavelengths of light [33,34]. The most thoroughly investigated phytochromes are those from *Arabidopsis thaliana*, which normally function to modulate seed germination and shade avoidance [35]. One such protein is phytochrome B (PhyB) which undergoes a conformational change upon exposure to light of visible red wavelengths (∼650–670 nm) to heterodimerize with the transcription factor phytochrome-interacting factor 3 (PIF3). Unlike other photosensitive proteins, this dimerization can be reverted through exposure to longer wavelengths of light (∼700–750 nm), which induces monoisomerization of PhyB and releases PIF3, allowing for very precise control of protein activity [32,36].

#### Cryptochromes

Also commonly found in plants, cryptochromes (Cry) are plant photosensors that absorb blue light, the most well studied of which, Cry2, heterodimerizes with the cryptochrome-interacting basic helix–loop–helix 1 (CIB1) transcription factor. Cryptochrome proteins have a C-terminal domain required for signal transduction and, like phytochromes, require a flavin adenine dinucleotide (FAD) chromophore cofactor, which binds to an N-terminal DNA photolyase homology region (PHR) [37,38]. Since the discovery of Cyr2 and its ability to heterodimerize with CIB1, this system has been adapted to circumvent the need for exogenous chromophore addition [37]. With this improved system, Kennedy et al. [37] induced dimerization of Cry2–CIB1 on a sub-millisecond timescale (in under 300 μs), although the reverse process took minutes to complete. Nevertheless, this improved system has since been used in the study of a number of different cellular processes in model organisms. One field in which the Cry2–CIB1 system has been used successfully both *in vitro* [39] and *in vivo* [40] is the study of phosphoinositide signalling. This was achieved by Cry2–CIB1-mediated recruitment of a phosphoinositide phosphatase catalytic subunit responsible for the conversion of PI(4,5)*P*_2_ into PI(4)*P* to the plasma membrane in a light-dependent manner. Using this approach, Guglielmi et al. [40] were able to study complex morphological changes and interactions that occur within defined timescales during *Drosophila* embryogenesis. The recruitment of the catalytic subunit to the plasma membrane within seconds of blue light illumination was sufficient for quick depletion of PI(4,5)*P*_2_ which, given the role of phosphoinositides in regulating actin polymerization, allowed control over cell contractility and facilitated the study of cell–cell interactions, force transmission and changes in tissue geometry [40].

The use of cryptochromes for conditional dimerization has since spawned a host of methods utilizing the interaction between Cry2 and binding partners such as CIB1. One such method, known as light-activated reversible inhibition by assembled trap (LARIAT), utilizes light-mediated heterodimerization to reversibly sequester target proteins into multimeric complexes in mammalian cells, by engineered interactions with multimeric proteins (Figure 1D, Table 1) [41]. Lee et al. [41] developed the LARIAT technique by fusing Ca^2+^/calmodulin-dependent protein kinase IIα (CaMKIIα) protein, which self-assembles into a 12-subunit oligomer, to CIB1. Upon blue light stimulation, CIB1 interacts with Cry2 and forms clusters through interconnections between CIB1-conjugated CaMKIIα multimeric proteins. Through this method of optogenetic trapping, Lee et al. [41] were able to induce cluster formation with high spatiotemporal precision in HeLa cells within 30 s of illumination, with cluster disassembly occurring within minutes of light withdrawal. Lee et al. [41] also found that the extent of clustering was correlated with the intensity or number of light pulses administered, suggesting that it may be possible to quantitatively control clustering simply by varying light conditions for more intricate control of protein dynamics. This approach can also be used to inactivate GFP-tagged proteins, without the need to add an additional protein tag, through the use of anti-GFP nanobodies. To demonstrate this approach, Lee et al. [41] trapped a number of different GFP-tagged proteins into complexes through interactions with a CIB1-conjugated anti-GFP nanobody to acutely disrupt proteins involved in fast-acting processes such as membrane retention and spindle formation.

A recent example that displays the potential of the LARIAT approach is its application to the study of intracellular membrane trafficking. Here, Nguyen et al. [42] developed a system whereby intracellular membranes can be rapidly and reversibly sequestered into complexes via Cry2-induced aggregation of CIB1-conjugated GTPases. Using diverse Rab GTPases as membrane markers, it was possible to access specific intracellular membrane compartments such as the Golgi and endoplasmic reticulum [42]. This approach makes it possible to dissect the spatiotemporal functions of intracellular membranes in a variety of processes such as receptor transport, intracellular signalling from endosomes, protein sorting and secretion.

**Figure 2:**
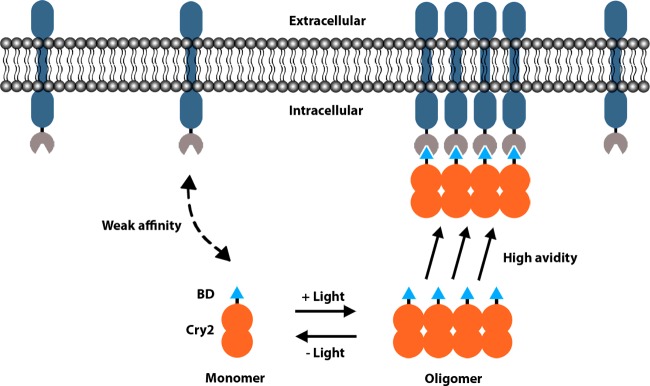
Clustering indirectly using cryptochrome 2 (CLICR) In the dark un, induced state, monomers of Cry2 fused to a receptor targeting BD (Cry2–BD) exist in an unclustered state and therefore have a weak affinity for the target receptor. Upon blue light stimulation, Cry2–BD molecules oligomerize, increasing the local concentration of BD and conferring a high avidity for the target receptor. These oligomers undergo membrane translocation and cluster target transmembrane receptors.

It is also known that many plant photosensors, including Cry2, are capable of forming aggregates upon light stimulation [43,44]. For example, Wend et al. [45] demonstrated the ability of Cry2 to dimerize C-Raf and activate its kinase activity in a light-inducible manner, functionally separating C-Raf from upstream growth factor signalling, enabling a more controlled approach to study dynamic downstream effects on target protein phosphorylation and cell signalling. Interestingly Wend et al. [45] also tested the ability of C-Raf-Cry2 to dimerize with CIB1-bound C-Raf and found a weaker activation of C-Raf, which they suggest may be due to a difference in stoichiometry when the larger Cry2 molecule binds to the much smaller CIB1 domain. Another use of Cry2 dimerization is in a technique called clustering indirectly using cryptochrome 2 (CLICR), which involves the clustering of transmembrane receptors to activate signal transduction (Figure 2). This is achieved by indirect clustering of Cry2 bound to a receptor-binding domain (BD); high local concentrations of the BD then serve to cluster endogenous receptors leading to signal activation [46]. An N-terminal src-homology 2 (SH2) domain, which binds receptor tyrosine kinases (RTKs) and the phosphotyrosine-binding-like F3 domain from talin, which binds β3-integrin were shown to be effective BDs [46], suggesting the method could be modified to target a wide range of transmembrane proteins. However, the selectivity of such tools needs to be empirically validated for each system.

#### Light–oxygen–voltage domains

An alternative approach to light-induced dimerization involves the use of light–oxygen–voltage (LOV) domains from *Avena sativa* phototropin 1 (Table 1). LOV domains contain a C-terminal α-helix (Jα helix) which, upon light illumination and excitation of a flavin cofactor within the LOV domain, undergoes a large conformational change and unwinds [47]. This light-induced structural change allows for the control of protein activity through allosteric regulation of proteins containing these LOV domains (Figure 1E). One example of how LOV domains have aided the study of protein function is the application to the study of cell motility [48,49]. Wu et al. [48] fused Rac1 to a LOV domain, which, in its native α-helix state, blocked Rac1 interactions. This photo activatable Rac1 (PA-Rac1) could then be reversibly and repeatedly activated in precise cellular locations by illumination with blue light, producing localized cell ruffling and protrusions. Localized Rac1 activation was also able to promote directed cell motility [48]. PA-Rac analogues have since been used in further *in vivo* cell migration studies, showing for instance that Rac activation is sufficient for polarization of the border cells in *Drosophila* oogenesis and that the directionality of the subsequent migration of these cells during egg chamber development is dependent on Rac levels [50]. Although PA-Rac has been used successfully in a number of studies, the shift between the wound and unwound Jα helix states upon illumination is less than ideal, with at best a 10-fold shift towards the unfolded state upon light irradiation [51]. Through the identification of mutations that stabilize both the wound and unwound Jα helix states, Strickland et al. [52] modified the LOV system and reduced the proportion of unwound Jα helix in the dark state to make the switch between light and dark states more defined and increase the dynamic range of the system as a whole, with up to a 70-fold shift in Jα helix state after exposure to light.

One example that takes advantage of the high spatial and temporal precision that can be achieved using LOV domains is the control of RTK activation. RTKs are a family of cell-surface receptors that respond to growth factor and hormone signals to regulate a variety of cell behaviours, and have previously proven difficult to study due to the rapid rates of receptor biosynthesis and degradation that can occur. Grusch and colleagues [54] used LOV-domain-mediated dimerization of mutant RTKs, insensitive to endogenous ligands, to induce transphosphorylation and therefore receptor activation on a timescale shorter than that of receptor synthesis/degradation. Through this approach they were able to mimic the cell behaviours induced by endogenous growth factors to provide control over cell signalling on the minute timescale, with diverse cellular responses in different cell types pointing to the involvement of different adapter proteins or feedback mechanisms [54].

Light-based methods for induced protein complex formation and control of protein activity offer a powerful solution to many of the drawbacks that come with chemical-based approaches while maintaining versatility. Although light-based methods require laser excitation to stimulate photoactivatable protein modules, the wavelengths of light used generally fall within the same range as those used for conventional fluorescence imaging, meaning cytotoxic effects are minimal and these approaches have therefore been applied successfully to both *in vivo* and *in vitro* studies [55]. The benefits of optogenetic approaches over the more traditional well-studied small-molecule approaches suggest that, with further development, these tools will be invaluable in the use of complex formation for protein inactivation or sequestration in the study of fast-acting cellular processes.

## PROTEIN CLEAVAGE/SPLICING

**Figure 3:**
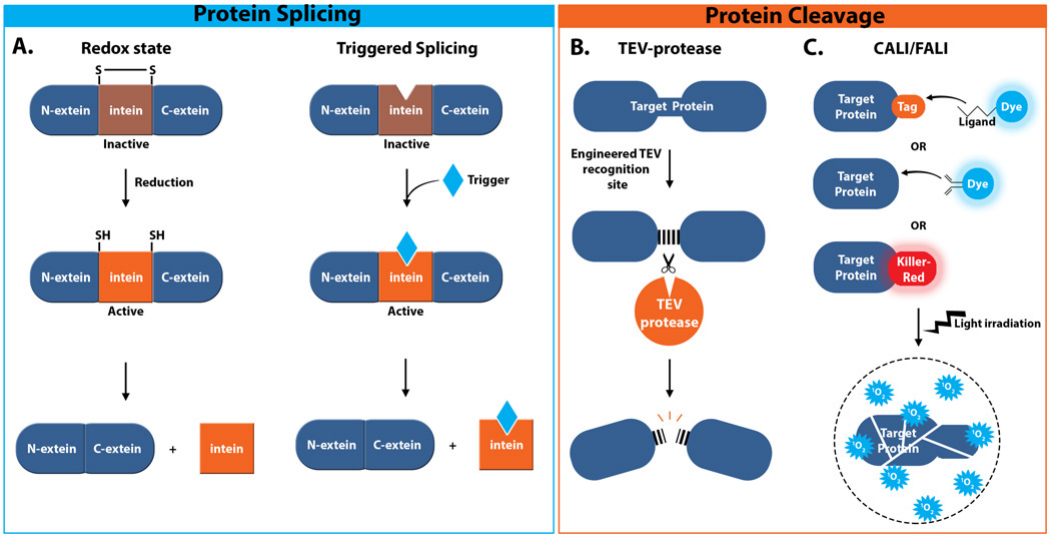
Illustration of methods for conditional protein splicing or protein cleavage to (in)activate target proteins (**A**) Illustration of conditional protein *cis*-splicing induced by activation of an intein through a change in redox state or via a trigger, which may be addition of a small molecule, as depicted here, a change in pH, temperature or irradiation with a specific wavelength of light. Protein *trans*-splicing is also possible, whereby dimerization domains can be used to reassociate split intein fragments upon addition of a trigger. (**B**) Illustration of TEV protease-mediated cleavage of a TEV recognition site engineered within a protein of interest leading to protein inactivation upon induction of TEV protease expression. (**C**) Schematic representation of CALI/FALI induced by addition of a dye-conjugated ligand or antibody, or via genetically encoded methods involving photosensitizers such as KillerRed, eGFP, miniSOG or SuperNova. Upon irradiation with a specific wavelength of light these produce ROS (^1^O_2_) leading to inactivation of proteins in the immediate vicinity.

Another common strategy for the inducible control of protein activity is to induce physical changes in protein sequence through the endogenous process of protein cleavage or splicing. As with methods for inducible protein complex formation, protein cleavage/splicing can be engineered to allow induction via a number of different mechanisms including both small-molecule-based and light-based approaches (Figure 3, Table 2). However, the mechanisms used to induce protein cleavage/splicing are often interchangeable, allowing these methods to be adapted to a wider range of systems and biological questions.

**Table 2:** Summary of methods for conditional control of protein splicing/cleavage

			Can be induced by			
Technique	Protein disruption	Timescale	Small molecule/hormone	Light	Temperature	pH
Intein-mediated protein splicing	Inactivation via protein splicing	h	✓	✓	✓	✓
Tobacco etch virus (TEV) protease cleavage	Inactivation via protein cleavage	min	Promoter-dependent			
Chromophore-assisted light inactivation (CALI)	Inactivation by ROS	Often <1 s	✗	✓ e.g. Malachite Green: 616–624 nm KillerRed: 540–580 nm	✗	✗
Fluorophore-assisted light inactivation (FALI)	Inactivation by ROS	<10 min	✗	✓ e.g. FITC 493–518 nm	✗	✗

### Intein-mediated protein splicing

One method that allows inducible control of protein activity uses the endogenous post-translational mechanism known as intein-mediated protein splicing (Table 2). With this method, intervening polypeptides known as inteins are used to catalyse their own removal from the flanking polypeptides, or exteins, which are subsequently joined back together. Inteins are typically removed in a four-step process involving conversion of the peptide bond linking the N-terminal extein to an ester or thioester bond and transfer of the N-extein to the C-extein by transesterification. The resulting branched ester is then resolved by asparagine cyclization followed by conversion of the newly formed ester bond linking the two exteins into an amide bond and hydrolysis of the C-terminal aminosuccinimide of the excised intein [56,57]. Inteins are used in biotechnology for a number of different applications, including the control of protein expression or modification, post-translational processing and also protein labelling [57], but perhaps the most valuable application in terms of studying protein function is to facilitate the control of protein activity. Since inteins are extensively reviewed elsewhere [57–59], so we will not discuss their use further here, except to say that they have been engineered to allow conditional protein splicing (CPS), such that the splicing process is induced by the activation of an intein through reduction or the addition of a trigger such as light, temperature, pH or the addition of a small molecule (Figure 3A) [57,60]. These systems have been used successfully both in cultured cells and in living animals to interrogate protein function, although they have not been widely adopted for this purpose perhaps because of their intrinsic lack of reversibility.

### TEV cleavage

One common method for inducible protein cleavage as a mechanism to control protein activity exploits the ability of the tobacco etch virus (TEV) protease to cleave a highly specific seven-amino-acid recognition sequence (E-X-X-Y-X-Q-G/S) with high efficiency (Figure 3B, Table 2) [61–63]. TEV is commonly used as a mechanism for the cleavage of fusion proteins to remove protein affinity tags prior to further protein analysis [64]; however, this system has also been applied to the control of protein activity both *in vivo* and *in vitro*. Through genetic modification, the TEV recognition sequence can be engineered into a protein of interest to allow inducible protein cleavage and inactivation when in the presence of TEV. TEV techniques have previously been demonstrated in budding yeast to provide evidence of a role for separin in anaphase initiation [65] and has since been applied to the study of proteins in both *Drosophila* cell culture and live embryos [66,67]. For example, to show that TEV was able to effectively and specifically cleave a protein containing the recognition sequence in live *Drosophila* embryos, Harder et al. [66] expressed the protein Megatrachea (Mega), a *Drosophila* claudin protein localized to membrane compartments of ectodermal cells, containing an artificial TEV protease cleavage site (TEV_pcs_) and YFP (Mega-TEV_pcs_-YFP). Upon TEV expression using the Gal4/UAS system, Mega-TEV_pcs_-YFP no longer showed the correct YFP localization indicating that the YFP had been cleaved from the Mega fusion protein. Harder et al. [66] went on to adapt this system to allow induction of TEV expression at different stages of embryo development by putting TEV under the control of the heat-shock protein 70 (hsp70), thus generating a mechanism for the temporal control of TEV-mediated protein cleavage. Using this temporally controlled system, they were then able to induce cleavage of a Mega-TEV_pcs_ construct leading to truncation of the Mega protein and subsequent degradation of the truncated protein [66]. Clearly, a key factor in determining the timescale of TEV-mediated cleavage is the promoter from which TEV is expressed. Changes in temperature using the heat-shock protein are capable of inducing TEV expression and protein cleavage in *Drosophila* pupae ∼3 h after a 45 min heat shock [67], whereas rapamycin-induced expression in mammalian cells induces cleavage within 150 min [68]. This may make the approach unsuitable for the study of some processes that operate on a short timescale.

One disadvantage of TEV is that it readily cleaves itself at a specific site to yield a truncated enzyme with greatly reduced activity [69,70]. There have therefore been a number of iterative changes made to TEV protease to adapt the protease for more diverse applications, for example a TEV mutant has recently been designed specifically to be active in the secretory pathway [71]; various other TEV mutants offer the same recognition site cleavage, but an increased stability and reduced auto-cleavage activity [69]. Although TEV offers highly specific and efficient cleavage, *a priori* knowledge about protein composition is required to choose a position where the TEV recognition site can tolerably be inserted that will inactivate the target protein while reducing the possibility that the resulting protein fragments will retain function or even have novel functions of their own. There is also an optimum level of TEV protease expression at which cleavage occurs but background activity is minimalized; this level is likely to depend on both the variation of TEV protease and the system in which it is applied so would need to be considered during experimental design [68].

The timescales and spatiotemporal resolution of both intein-mediated protein splicing and TEV cleavage are dependent on the engineered mechanism of induction. Although the ability to customize these techniques allows them to be applied to a wide range of systems and cellular processes, the fastest acting methods of protein cleavage/splicing with the highest spatiotemporal resolution are again those induced by light.

### CALI/FALI

One method for light-inducible protein cleavage applied to the study of protein function is chromophore-assisted light inactivation (CALI) (Table 2). Chromophores are photosensitive groups, often responsible for the colour of organic molecules, which produce highly reactive free radicals such as reactive oxygen species (ROS) upon illumination with a specific wavelength of light. Using the CALI approach, a chromophore-tagged protein of interest is inactivated through mild illumination for a period of time sufficient to induce generation of ROS and induce protein cleavage of proximate proteins, but short enough to ensure that the ROS act within a defined radius [30–40 Å (1 Å=0.1 nm)] to minimize off-target effects (Figure 3C) [72]. The specificity of CALI approaches is determined by the short half-life of the free radical species, which ensures that only proteins within a radius of 1.5–6 nm relative to the chromophore are affected [72–74]. After free radical generation, proteins are typically inactivated within 1 s, which, combined with laser irradiation of micrometre accuracies, allows for high spatial resolution and highly controlled protein inactivation [74].

Originally, CALI approaches used an antibody-based mechanism to attach a chromophore, such as the dye Malachite Green, to a protein of interest (Figure 3C). Fluorophores such as fluorescin isothiocyanate (FITC), which are more efficient at ROS production, were later employed in a similar approach called fluorophore-assisted light inactivation (FALI) [75]. However, the need for microinjection of a dye-labelled non-function-blocking antibody specific to the protein of interest limited the widespread application of these approaches. Subsequent methods made use of genetic modification to label proteins with a generic tag that can then be fluorescently labelled through extracellular addition of a specific reagent (Figure 3C). Proteins tagged with one or two small tetracysteine (TC) motifs will specifically bind to the membrane permeable biarsenical dye resorufin-based arsenical hairpin binder (ReAsH) or the fluorescein-based arsenical hairpin binder (FlAsH). For example, Marek and Davis [76] used FlAsH labelling to visualize synaptotagmin I (Syt I) at the neuromuscular junction (NMJ) in late-stage live *Drosophila* larvae. Through photo-inactivation they were able to inactivate Syt I within seconds and provide supporting evidence for a model, previously based on genetic data alone, in which Syt I plays a role post-vesicle docking to mediate vesicle fusion and calcium-dependent transmitter release [76].

Although dyes are added extracellularly in ReAsH/FlAsH based CALI/FALI approaches, the application of these techniques *in vivo* is limited by the difficult task of achieving sufficient uptake in live animals and also the inability to spatially control the production of ROS and limit it to particular cells or subcellular compartments [77]. There is also the problem of non-specific binding of the membrane-permeant dyes. For CALI/FALI to become a more widely used technique, there was therefore a need for a system that sidestepped the requirement for exogenous addition and could be encoded completely through genetic manipulation. There has been some limited success using eGFP, a tag commonly used to study protein localization and function. For example, CALI illumination of GFP–myosin II was shown to result in unequal-size daughter cells during asymmetric cell divisions in a *Caenorhabditis elegans* Q neuroblast cell lineage [78]. It is, however, believed that the chromophore within the GFP structure is protected by the outer shell meaning the generation of free radical species upon illumination is restricted and therefore GFP holds a low phototoxicity [77,79].

#### KillerRed

The first example of a successful genetically encoded CALI reagent, with a 1000-fold increase in phototoxicity compared with GFP, is the GFP-related red fluorescent protein KillerRed, developed from the non-fluorescent red chromoprotein anm2CP of *Hydrozoa* jellyfish (Figure 3C) [79]. Although it was originally unclear why there was such an improvement in phototoxicity, a study into the structure–function relationship of KillerRed by Pletnev et al. [80] provided crystallographic data revealing unique structural features that may facilitate ROS generation. KillerRed has been used in a number of applications in biological research, such as the control of protein activity in the study of neuronal development in model organisms [81], and has been used to induce cell-specific killing of eukaryotic cells in culture via mitochondrial or membrane-targeted KillerRed [79]. In this regard, KillerRed has also proved to have uses in medicine; one recent and exciting application of KillerRed was to the experimental cancer treatment known as photodynamic therapy (PDT), which aims to use photosensitizers to selectively kill tumour cells through ROS generation upon laser illumination [53,82]. Following subcutaneaous injection of KillerRed-expressing *Escherichia coli* (KR-*E. coli*) into mouse xenograft models of human carcinoma cell lines the Terekawa laboratory [82] monitored the intensity and spread of fluorescence through the tumour cells. After 24 h the KR-*E. coli* spread throughout the whole tumour and were subsequently irradiated with orange light (540–580 nm) to induce ROS production. The generation of ROS led to necrosis and tumours gradually disappeared to leave healed skin after just 1 week, demonstrating the ability of KR-*E. coli* to kill cancer cells originating from humans. Although there are many questions still to be answered before this technique is applicable to humans, these results provide an insight into the capabilities of genetically encoded CALI approaches.

KillerRed has proved to be an exciting solution to the difficulties associated with previous CALI approaches. However, one limitation is its tendency to homodimerize, which can potentially interfere with protein function [77,79]. There have been a number of other novel photosensitizers discovered since, such as SuperNova, a monomeric form of KillerRed [83], and the fluorescent flavoprotein mini Singlet Oxygen Generator (miniSOG), which can also be used to generate an insoluble deposit of singlet oxygen species that can be stained for visualization using electron microscopy [84]. More recently, the toolkit of phototoxic proteins was expanded further with the addition of KillerOrange, an orange mutant version of KillerRed that results in ROS formation upon illumination with either blue or green light, meaning it can be used in combination with KillerRed or other photosensitizers activated by different wavelengths of light [85].

CALI/FALI approaches enable specific protein inactivation through phototoxicity and can act with high spatial resolution through the ability to express tagged proteins in specific cells and trigger inactivation at a subcellular level (Table 2). The expansion of the phototoxic protein toolbox should also allow for the creation of more intricately controlled systems in which different proteins can be inactivated at different time points or in different cell populations, which will form a useful tool both for fundamental research and for potential medical applications. However, CALI/FALI-based methods suffer from a number of limitations, including the requirement for either exogenous ligand addition or the inclusion of a fairly large protein tag that may interfere with protein function. Importantly, there is also potential for off-target effects on proteins in close vicinity to the ROS generator, challenging the specificity of these approaches. For example, Guo et al. [86] found that the inhibition of calcium ion currents, mediated by the class C G-protein-coupled receptor (GPCR) mGluR8a, was greatly attenuated following FALI inactivation. Although initial results were consistent with acute inactivation of mGluR8a, Guo et al. [86] also reported collateral damage to proximal proteins with no overt link to pathways of GPCR signalling. These factors have limited the adoption of such methods to study protein function and cannot be overlooked when using techniques involving phototoxicity for protein inactivation.

## PROTEIN STABILITY/DEGRADATION

**Figure 4:**
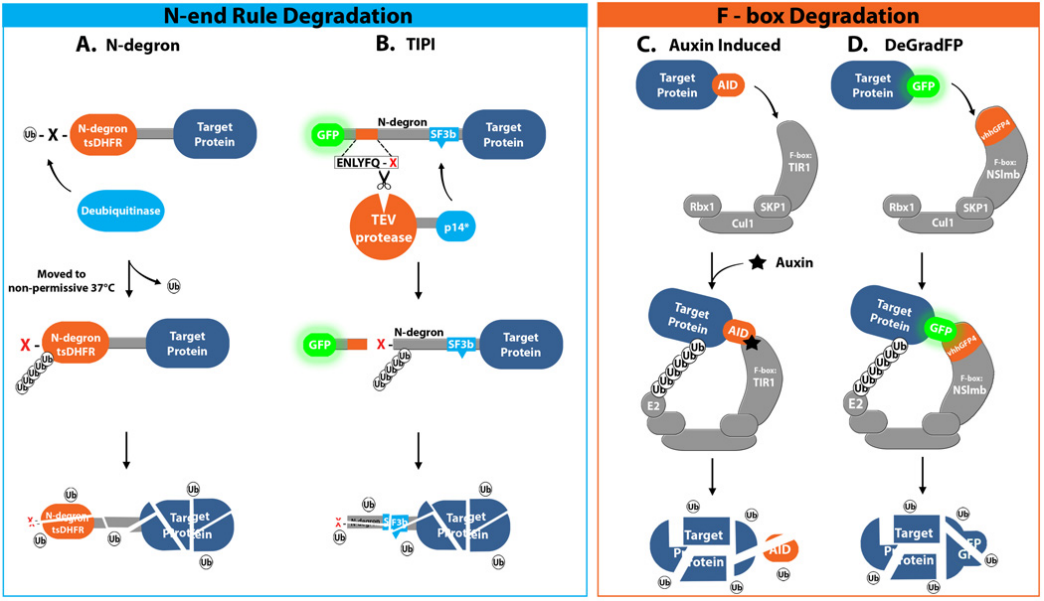
Illustration of methods for inducible protein degradation divided into those involving the N-end rule or F-box-based pathways (**A**) N-end degron method, involving the exposure of an unstable N-terminal amino acid which can be induced by a number of mechanisms including temperature- or small-molecule-based mechanisms. This unstable end is subsequently targeted for polyubiquitination by an E3 ligase, such as Ubr1P in yeast, and degraded via the proteasome pathway. (**B**) TIPI which utilizes TEV protease-mediated cleavage of a seven-amino-acid TEV recognition site to reveal an unstable N-terminal amino acid, subsequently targeted for proteasome-mediated degradation as for the N-end degron approach. The efficiency of TEV cleavage is increased by the inclusion of a short SF3b155^381–424^ domain downstream of the TEV recognition site, which binds to a mutant version of the human spliceosome subunit 14 (p14*) bringing p14*-TEV to the recognition site. (**C**) Illustration of the mechanism for auxin-induced degradation of target proteins tagged with an AID. Upon addition of auxin, the AID-tagged protein is recruited to an engineered E3 ubiquitin ligase SCF complex, containing the TIR1 F-box protein from plants, which binds target proteins in the presence of the plant hormone auxin. Expressing TIR1 in non-plant cells is enough to result in formation of the SCF complex which then binds AID-tagged proteins in an auxin-dependent manner, leading to polyubiquitination and degradation via the proteasome. (**D**) Schematic illustration of the deGradFP method for inducible protein degradation. The GFP-tagged target protein is recruited to an engineered SCF complex containing the F-box protein NSlmb conjugated to an anti-GFP single-chain antibody (vhhGFP4). Target protein is polyubiquitinated via the recruited E2 ubiquitin ligase and subsequently degraded via the endogenous proteasome machinery.

Although protein cleavage or splicing can often lead indirectly to protein instability and degradation, it is likely that this will occur after some delay depending on the half-life of the protein. It is also possible for the resulting protein fragments to retain function or bind other proteins and perform independent functions of their own, potentially generating a more severe phenotype than simple protein knockout alone. It therefore follows that a more thorough and interpretable approach for complete removal of proteins from cells is to target them directly for degradation by the endogenous cellular degradation pathways (Figure 4, Table 3). Although the direct degradation of target proteins means these methods are technically irreversible and somewhat limited in their application, in most cases protein levels return to normal following relief of the degradation stimulus and thus these methods can still provide a useful tool for studying protein function [9,10,87]. Methods for inducible protein degradation generally involve the proteasome pathway for protein degradation, in which ubiquitin is transferred from the E1 ubiquitin-activating enzyme to the E2 ubiquitin-conjugating enzyme and subsequently to a lysine residue within the target protein in a transfer facilitated by the E3 ubiquitin ligase [88]. This process is repeated to polyubiquitinate the target protein until it has a sufficient number of ubiquitin molecules for targeted degradation by the proteasome [88].

**Table 3:** Summary of methods for conditional control of protein degradation

			Can be induced by			
Technique	Protein disruption	Timescale	Small molecule/hormone	Light	Temperature	pH
N-end degron	Unstable N-end amino acid ubiquitination	min/h, sometimes <30 min	✓ e.g. Methotrexate	✗	✓ e.g. 37–42°C	✗
TEV protease-mediated induction of protein instability (TIPI)	Unstable N-end amino acid ubiquitination	min/h	Promoter-dependent			
Auxin-induced degradation (AID)	F-box-induced ubiquitination	<30 min	✓ Auxin	✗	✗	✗
Proteolysis-Targeting Chimaeras (PROTACs)	Direct targeting to E3 ligase complexes	min/h	✓ PROTAC	✗	✗	✗
deGradFP	F-box induced ubiquitination	min/h	Promoter-dependent			

### N-end degron

A common strategy for the direct induction of protein degradation utilizes the UBR1 E3 ligase pathway and the N-end rule (Table 3), which states that the half-life of a protein is determined by both the accessibility of lysine residues, for ubiquitination, and the identity of the amino acid at the N-terminus [89,90]. Varshavsky [90] demonstrated this principle through cleavage of ubiquitin, via a yeast deubiquitinating protease, from a fusion protein expressed in yeast, containing the 5′ end of a lacI linker followed by β-galactosidase (β-gal), resulting in the exposure of a new N-terminal amino acid. The half-life of β-gal following cleavage could then be vastly altered by simply changing the exposed amino acid residue. For example, β-gal with an N-terminal arginine or phenylalanine residue had a half-life of ∼3 min, whereas N-terminal methionine or valine resulted in a half-life greater than 20 h [89]. This method of degradation is conserved from bacteria to higher eukaryotes, and means proteins tagged with the unstable lacI degron can be targeted for degradation within minutes [90]. However, this strategy is not inherently inducible and therefore requires modification for the conditional control of protein degradation.

One of the first examples of an inducible N-end degron involved the use of a temperature-sensitive dihydrofolate reductase variant (tsDHFR) where an N-terminal destabilizing arginine was only exposed at non-permissive temperatures [91]. By fusing the tsDHFR to the N-terminus of a target protein, degradation can be induced by a switch to the non-permissive temperature of 35°C, exposing the N-terminus at which point the N-end rule takes effect (Figure 4A). For example, this system has been used successfully in *Drosophila* to inducibly polyubiquitinate an eGFP reporter protein at the neuromuscular synapse following a 30 min heat shock at 35°C, in order to track the degradation of polyubiquitinated proteins [92]. By tracking the degradation of the eGFP, Speese et al. [92] showed that ubiquitinated presynaptic proteins are not removed from the synaptic terminal but rather undergo local proteasome-mediated degradation at pre-synaptic sites. In addition to examples from *Drosophila*, this technique has also been used successfully to characterize many essential proteins in budding yeast [93]. However, such techniques are generally limited to systems that can survive the required temperature changes and also to proteins that retain function with the required N-terminal modification. Despite this, the approach has since been used successfully in chicken DT40 cells, in which the method was first tested using a tsDHFR degron fused to eGFP. Upon transfer of the cells to the non-permissive temperature of 42°C, the protein was rapidly depleted to ∼10% of initial levels within 90 min leading to undetectable levels after 120 min [94]. Moving cells back to the permissive temperature of 35°C resulted in an efficient recovery to the pre-depletion level within 150 min. Su et al. [94] then used the approach to deplete RAD51, finding that RAD51, which plays an important role in homologous DNA recombination (HDR), does not stop DNA synthesis but causes cell cycle arrest in G_2_, suggesting HDR becomes important at G_2_. This, along with the many other applications of tsDHFR-based approaches, show that, although this method is limited in its potential applications, tsDHFR can still provide a useful tool in the study of protein function.

In addition to tsDHFR, a small- molecule-controlled version of dihydrofolate reductase (DHFR) has also been engineered, for which the drug methotrexate (MTX) regulates stability of the N-terminus. Although the presence of MTX fails to inhibit recognition and therefore polyubiquitination of tsDHFR by E3 ligase, the stable high-affinity interaction between MTX and DHFR impedes protein unfolding and prevents degradation by the proteasome [95,96]. This system was demonstrated in both yeast and mammalian cells in culture, although the occupancy of the proteome by the MTX–DHFR complex is likely to inhibit degradation of other cellular proteins leading to off-target effects [97]. Also, DHFR is required for the production of tetrahydrofolate, which is subsequently required for the synthesis of purines, thymidylate and several amino acids [98]. The inhibition of DHFR by MTX therefore interferes with the synthesis of DNA, RNA and even proteins, meaning MTX is undesirable as a regulatory small molecule for exogenous addition.

### TEV protease-mediated induction of protein instability (TIPI)

An alternative way in which the N-end degron system can be made inducible, and more widely applicable, is through the use of TEV in a technique called TEV protease-mediated induction of protein instability (TIPI) (Table 3). This technique combines TEV with the N-end rule, whereby TEV cleaves a recognition sequence engineered into a cryptic N-degron, attached to the N-terminus of a target protein, to reveal an unstable N-end amino acid (Figure 4B) [99]. According to the N-end rule, this unstable amino acid targets the protein for polyubiquitination and degradation via the UBR1 E3 ligase pathway [89,90]. TEV protease has previously been shown to allow degeneracy within its recognition sequence and is particularly flexible to changes at position 7, the amino acid residue that forms the N-end following TEV cleavage [63]. TEV protease can therefore cope with the incorporation of an amino acid that induces degradation following cleavage via the N-end rule. Taxis et al. [99] first developed the TIPI approach by designing a construct containing a reporter followed by a TEV protease recognition site, N-degron and SF3b155^381–424^ termed Reporter-TDegX-tag, where X represents the amino acid at position 7 which becomes the new N-terminal amino acid upon cleavage. The inclusion of a relatively short SF3b155^381–424^ domain allowed for more efficient cleavage as its binding to a mutant version of the human spliceosome subunit 14 (p14*) recruited p14*-TEV to the recognition site (Figure 4B). Taxis et al. [99] initially demonstrated this system in yeast using a GFP-TDegX-Don1p fusion protein, with p14*-TEV expression driven by the Gal1 promoter, monitoring cleavage via the release of GFP and testing the effect of the amino acid at position X on TEV cleavage efficiency and protein half-life. Phenylalanine or asparagine was found to provide optimal conditions for both TEV cleavage and rapid degradation of the target protein Don1p. The effectiveness of TIPI was shown by using the approach to deplete several different proteins in yeast, obtaining phenotypes correlating to those observed via genetic knockdowns [99].

The potential of TIPI as an approach for conditional degradation is yet to be realized; however, its power and versatility has recently been demonstrated in some alternative applications. For instance, TIPI has been further modified (mTIPI) to facilitate production of recombinant proteins; it does this by blocking endocytosis in yeast and combating a common problem whereby highly active endocytosis in protein expression systems reduces the overall protein yield [100]. For conditional control this method simply requires expression of the p14*-TEV fusion protein, which could be induced via the same methods as for TEV cleavage, including temperature, pH, small molecule addition or the Gal4/UAS system, making it a versatile tool for inducible degradation and the study of protein function both *in vivo* and *in vitro*.

### Auxin-induced degron

Another way in which proteins can be directly targeted for degradation by the ubiquitin–proteasome machinery is via F-box proteins, which bind target proteins and recruit the Cullin–RING complex, also called the SCF complex consisting of Skp1, Cullin and F-box, to ubiquitinate the target protein [101]. Eukaryotes contain multiple forms of SCF, whereby the F-box protein conveys specificity towards different target proteins. One F-box particularly suited to small-molecule-induced control of protein degradation is the transport inhibitor response 1 (TIR1) protein, which binds target proteins in the presence of the plant hormone auxin and has a highly conserved interaction with the E3 ligase protein Skp1 (Figure 4C) [102,103]. This is commonly referred to as an auxin-inducible degron (AID) system (Table 3), and was initially shown to be applicable to most eukaryotes (excluding plants), including budding yeast and cell lines derived from human, mouse, hamster, monkey and chicken [104]. The AID degron consists of IAA17, also known as AXR3, from *Arabidopsis thaliana* and when expressed at either the C- or N-terminus of GFP in budding yeast also expressing AtTIR1, under the control of the galactose-inducible *GAL* promoter, the SCF–TIR1 complex was able to assemble and degrade GFP to less than 3% of initial levels within 30 min of auxin addition [104]. This approach was successfully used to degrade several essential nuclear or cytoplasmic proteins in yeast. However, to apply the system to mammalian cells, it was first necessary to modify TIR1 to convey a higher thermostability and allow use at 37°C. This was achieved by sourcing TIR1 from the rice plant *Oryza sativa* (OsTIR1), which also provided an improvement over AtTIR1 for use in yeast [104].

This method has since been used successfully to degrade both nuclear and cytoplasmic proteins in *C. elegans* [105] and mammalian cells [106] to help identify the function of several different target proteins at specific time points of the cell cycle. Holland et al. [106] tested five differentially localized proteins, some of which were known to be incorporated into protein complexes. Four out of the five AID–YFP-tagged proteins expressed under doxycycline control (Plk4, CENP-A, TFR2 and cyclin B1) showed quantitative protein degradation within 80 min of auxin addition. Degradation of the other protein studied, H2B, occurred more slowly, within 3 h [106]. These results show that AID is capable of rapidly depleting proteins involved in stable complexes with relatively long half-lives; however, the time taken for depletion following induction can vary. Holland et al. [106] demonstrated the ability of AID to induce proteolysis with the same or very similar degradation kinetics at all phases of the cell cycle and also found that proteins reappeared almost immediately upon removal of auxin stimulus. To demonstrate the ability of AID to study protein function, Holland et al. [106] achieved rapid functional inactivation of BubR1, an essential component of the mitotic checkpoint, by depleting endogenous BubR1 protein with siRNA, replacing it with siRNA-resistant GFP-AID-BubR1 and inducing mitotic arrest through nocodazole addition. GFP-AID-BubR1 rescued the function of the depleted endogenous BubR1 and this rescue could be rapidly reverted through auxin-induced degradation of the GFP-AID-BubR1 fusion protein to produce a more complete null phenotype than mRNA depletion alone [106].

The AID approach has since been developed further to increase versatility through minimization of the degron size and the inclusion of a series of epitope tags to allow detection using fluorescence microscopy or commercially-available antibodies [107]. Morawska and Ulrich [107] developed a series of vectors for PCR-based genomic tagging strategies containing different iterations of the AID degron with epitope tag, allowing for both C- or N-terminal tagging and providing a range of selection markers which they then demonstrated through application to a series of different yeast proteins. Although these vectors increase the versatility and facilitate the use of the AID approach, individual proteins must still be considered on a case by case basis to design the most effective degron; it may even be necessary to test multiple iterations to ensure proteins retain function.

### PROTACs

Another chemical-based method for the specific degradation of target proteins by the endogenous ubiquitin–proteasome machinery is through the use of heterobifunctional small molecules known as Proteolysis-Targeting Chimaeras (PROTACs) (Table 3). PROTACs consist of one moiety that binds the target protein linked to an E3 ligase to directly recruit the protein for proteasome-mediated degradation. Initially developed to target disease-causing proteins for destruction, the first generation of PROTACs were based on large peptide motifs derived from known ubiquitin ligase substrates [108]. However, these were limited by high molecular mass, poor cellular uptake and potential metabolic instability [109,110]. Following a switch to small-molecule-based PROTACs, the last decade has seen a series of improvements to PROTAC technology, aided by the development of small ligands for a number of E3 ligases, including MDM2 (murine double minute 2), cIAP1 (cellular inhibitor of apoptosis protein 1), CRBN (cereblon) and VHL (von Hippel–Lindau protein) (as reviewed in [110]). Although these improved small- molecule PROTACs were able to successfully degrade target proteins, the overall uptake of this technique for the conditional control of protein degradation has been limited by a number of uncertainties, including PROTAC stability and E3 ligase binding affinity [109,110]. However, more recent advances in the field have provided a new generation of highly specific high-affinity low-molecular-mass PROTACs with the potential to expand the use of PROTAC technology [111–114].

For example, Bondeson et al. [111] used structure-guided approaches to develop low-molecular-mass (∼450 Da) high-affinity ligands for the Cullin-RING ligase 2 VHL E3 complex (CRL2^VHL^). Linked to small molecules that bind specific cellular targets, Bondeson et al. [111] were able to efficiently degrade specific proteins in cultured cell lines, including the serine/threonine kinase RIPK2, which is involved in innate immune signalling, and the oestrogen-related receptor α (ERRα), which is implicated in the regulation of various cellular metabolism pathways, with dose-dependent degradation and maximal degradation levels of >95 and 86% respectively. Bondeson et al. [111] also demonstrated this approach *in vivo* using a PROTAC targeting ERRα, reducing its levels by ∼50% and significantly reducing mouse heart and kidney tumours by >40%. Using a similar approach, Zengerle et al. [114] successfully designed potent PROTACs using optimized drug-like VHL ligands [115] and bromo- and extra-terminal (BET) bromodomain protein ligands to selectively degrade certain members of the BET protein family, including the epigenetic regulator BRD4 previously identified as a potential therapeutic target for acute myeloid leukaemia and ovarian cancer [114].

In addition to targeting proteins to the CRL2^VHL^ E3 complex, potent PROTACs have also been developed to utilize the interaction between immunomodulatory drugs (IMiDs), such as thalidomide, and the CRL4^CRBN^ E3 ligase. CRL4^CRBN^, together with an IMiD, forms a tertiary complex with the transcription factor Ikaros, resulting in its ubiquitination and degradation. This approach has since been used for the efficient and specific degradation of BRD2, BRD3 and BRD4 by attaching BET bromodomain protein ligands to an IMiD [112,113].

Although the principle behind PROTAC technology is not novel, there has been a recent surge of developments to generate a newer generation of more potent PROTACs, which address the limitations of previous iterations. These newer PROTACs offer greatly increased potency while retaining high specificity to their target proteins both *in vitro* and *in vivo*. It is also possible to modify this specificity through manipulation of the linker between the two PROTAC moieties [111,114]. The diversity of recently described examples shows how the PROTAC approach can work on different protein targets in a number of different systems. However, compared with small molecules used in other approaches to conditionally control protein dynamics, PROTACS are larger and more complex molecules and so may suffer limitations with respect to their pharmacokinetic properties.

### deGradFP

The F-box/SCF complex-based approach has also been utilized in the degrade GFP (deGradFP) method to specifically degrade GFP-tagged fusion proteins via an anti-GFP nanobody/F-box chimaera (Figure 4D, Table 3). A method to allow specific degradation of proteins tagged with GFP is desirable as GFP-tagged constructs already exist for many proteins and degradation can be easily monitored by the loss of fluorescence. The deGradFP method involves the engineered F-box fusion protein NSlmb-vhhGFP4, consisting of an F-box domain derived from the *Drosophila* protein Slmb and the single-domain anti-GFP antibody fragment vhhGFP4 which recognizes GFP and its close derivatives (Figure 4D) [116]. This method was initially demonstrated in *Drosophila*, where, with NSlmb-vhhGFP4 expression restricted to the posterior of early stage embryos by the Gal4/UAS system, an EYFP-tagged histone H2A variant (His2Av–EYFP) was rapidly depleted by the deGradFP system [116]. Caussinus et al. [116] used the *engrailed-Gal4* driver to express both NSlmb-vhhGFP4 and nuclear mCherry in embryos ubiquitously expressing His2Av–EYFP. Using mCherry levels as a reporter for the expression of NSlmb-vhhGFP4, His2Av–EYFP started to be degraded after ∼30 min following NSlmb-vhhGFP4 expression, with less than 10% of the maximum EGFP intensity remaining after ∼3 h [116]. Caussinus et al. [116] then went on to show the versatility of the deGradFP approach through the successful depletion of the cytoplasmic protein Spaghetti squash (Sqh), nuclear protein Apterous (Ap) and the transmembrane protein Crumbs (Crb) all of which were tagged with GFP, expressed in a null background and degraded upon induction of NSlmb-vhhGFP4 expression via the Gal4/UAS system. There were, however, a couple of cases in which the deGradFP was not effective against GFP-tagged target proteins. For example, E-cadherin/Shotgun (Shg) could not be degraded using this method, possibly as it exists in a large protein complex which may mean the GFP tag is not accessible to the vhhGFP4 antibody [116]. Also, NSlmb-vhhGFP4 was unable to induce degradation of GFP alone, perhaps as the small size of GFP prevents exposure to the SCF-recruited E2 enzyme and thus prevents poly-ubiquitination. It was, however, possible to degrade GFP containing a small nuclear localization signal via this method, so although it is possible that a minimum size limit exists, below which degradation does not occur, this limit must be very close to the size of GFP alone and should not greatly limit the versatility of the approach [116].

deGradFP has been proven to be a useful approach for the induced degradation of target proteins particularly in combination with RNAi knockdowns in order to generate a more effective depletion of protein levels [117,118]. More recently, a similar approach involving the modification of the E3 ubiquitin ligase adapter protein SPOP to alter target protein specificity was proposed [119]. By fusing an anti-GFP nanobody directly to a truncated SPOP adapter protein completely lacking its substrate-binding domain, Shin et al. [119] claim to have developed an approach that is more efficient than deGradFP, which simply involves an NSlmb deletion mutant for which the binding domain has been modified. This remains to be proved in terms of biological applications, but may offer an alternative in cases where deGradFP is not effective. deGradFP, and related methods, can in theory be adapted to allow knockdown of many endogenous proteins, providing high-affinity antibodies are available for target proteins [87].

### Ubiquitin-independent

Ubiquitin-mediated protein degradation is by far the most common strategy for control of protein degradation; however, it has previously been shown that localization to the proteasome is sufficient for degradation [120] and so it is worth mentioning here that there are also a handful of methods for the conditional control of ubiquitin-independent protein degradation. The most common of these is the C-degron; consisting of a 36 amino acid sequence from ornithine decarboxylase (ODC), this forms a bridged association with the proteasome, acting as both the recognition and degradation initiation signal [87]. An exciting use of this technique allows for light-induced protein degradation via the use of LOV2 domains [121]. Renicke et al. [121] designed a system in which the C-degron, fused to the C-terminus of the LOV domain, can be masked by the Jα helix under dark conditions, but exposed upon illumination with blue light via Jα helix unfolding, leading to ubiquitin-independent protein degradation. Although this provides an exciting alternative, ubiquitin-dependent methods remain the most widely used and well-studied methods for inducible protein degradation.

## CONCLUDING COMMENTS AND FUTURE PERSPECTIVES

Manipulation of genes, at the level of DNA or RNA, has proven to be a specific and immensely powerful way of understanding the roles of encoded proteins in their native cellular environment. However, distinguishing between the initial and steady-state consequences of gene disruption, especially *in vivo*, is often problematic. The new generation of tools and methods that are emerging to meet this challenge address the issue by offering both rapid and specific control of protein function. Different sets of tools can be employed across a range of timescales to challenge biological processes operating at the subcellular, cellular and multicellular level. Methods for conditional control of protein complex formation (Table 1), in particular, open the door to *in vivo* analysis of biochemical processes operating over short times (s/min), such as intracellular signalling cascades, which are initiated within seconds of receiving the stimulus. Furthermore, the ability to reversibly switch activities on and off enables the systematic perturbation of biochemical pathways, thereby revealing how information is processed from upstream stimulus to downstream effectors at each step and providing insights into rate-limiting components and feedback control [122]. Repetitive perturbation at different times has particular value in the dissection of biological systems where frequency variation, including oscillatory behaviours, encodes information [123,124]. Other techniques, including those that conditionally control protein splicing/cleavage or degradation, typically operate over minutes/hour timescales (Tables 2 and 3), but have proven utility in studying the molecular mechanisms of downstream events, such as changes in cell proliferation, differentiation, migration or adhesion, which operate over longer times.

It is important to note, as discussed in the sections above, that current methodologies for conditional perturbation of protein function are not without their technical limitations and researchers must weigh up whether the available tools offer the appropriate flexibility and precision for the desired experiment. One of the main considerations for researchers wishing to utilize the methods we have described is to decide which inducer they should use. Chemically induced methods have been the mainstay of the field for many years, but the use of chemical inducers is often restricted because of their promiscuous binding profiles, which can lead to off-target effects and cytotoxicity. As we have discussed, higher-affinity ligands might mitigate these effects, because the compounds can be used at lower concentrations, but typically suffer from not being reversible. More fundamentally, however, the inability to target some chemicals to specific subcellular localizations, combined with their relatively slow uptake in cells, with effects occurring in minutes to hours, make these methods unsuitable for the study of certain biological processes and *in vivo* models.

Recently, there has been a surge of activity to develop methods of induction using light. One of the key attractions of this approach is that perturbations can be both rapid and reversible, in a spatially resolved manner. Consequently, with light-dependent systems, should an activated protein diffuse out of the area in which it received the activating input, it will then switch off, preventing phenotypic outputs from losing their spatial resolution [55]. Correspondingly, there has been a great deal of focus on improving the properties of the light-sensitive domains used in these approaches, much in the same way that there have been iterative improvements made to fluorescent proteins for use in cell imaging. This will make it possible to tailor the perturbation dynamics. For instance, in the case of light-induced complex formation, a derivative of the Cry2 domain (Cry2-olig) with altered off kinetics may make it more suitable for sequestration and inhibition of protein activity [125]. A challenge to future efforts for improvement to such domains will be not just to identify variants that confer beneficial properties in isolation, but ones that retain additive effects of multiple genetic changes; this will require screening procedures that simultaneously optimize constructs against multiple parameters.

The ultimate goal of the approaches we have described is to understand the role of molecules in biological phenomena with quantitative precision. Quantitative *in vivo* biochemistry, however, requires measurement not only of the effects of perturbation on a given process, but also of the magnitude of the perturbation to the target protein in time and space. Although reliable molecular readouts might be available for the former, the latter may be somewhat harder to measure. Fluorescently labelled target proteins offer an attractive solution for methods relying on protein degradation, since fluorescence intensity can provide a measure of protein concentration with spatiotemporal resolution [126]. However, although quantification of other perturbations, e.g. protein cleavage or activation, may be straightforward in cell populations at fixed time points, measurements with single-cell resolution or in real time will be much more challenging to achieve. A number of advanced cell imaging approaches may make this possible, but these techniques are themselves technically demanding and may not be widely available to researchers who do not have access to specialized equipment. For instance, measurements of protein dynamics (e.g. with fluorescence cross-correlation spectroscopy, or raster image correlation spectroscopy) and protein proximity (e.g. with FRET and fluorescence lifetime imaging) can be employed to determine effects on protein complex formation [127], while specialist (e.g. FRET-based) reporters can measure, e.g., the activity of enzymes responsible for post-translational modifications [128]. Future developments will therefore have to consider appropriate strategies, not just to perturb protein function, but to simultaneously measure the extent of that perturbation along with the biological effect(s).

Concurrently, an ongoing challenge for technology developers is to make the techniques universally appealing and easy to employ. Ultimately, whether a technique is proven to be robust and fit for purpose will depend on uptake and testing by user communities. From a practical perspective, it may still take a significant investment of time to tailor an approach to the experimental system under investigation, despite efforts to make the available tools universally applicable. Prior knowledge of protein structure–function relationships may be required, for example, to guide the production of fusion proteins that retain normal activity and respond effectively to regulatory stimuli. These issues have inevitably limited the uptake of many of these techniques. Methods that utilize tools that are already widely employed, such as GFP-tagged proteins, are likely to be among the most popular in the short term because the methodologies can be rapidly deployed. For instance, deGradFP will no doubt be of particular interest to the *Drosophila* and zebrafish communities, which are creating transgenic libraries in which endogenous genes have been tagged with GFP [129,130]. Indeed, such collections may be the starting point for well-designed temporally controlled screens, to identify novel genes involved in developmentally regulated biological processes.

As the field continues to mature, an area of future development is likely to be how multiple techniques might be combined for improved spatiotemporal control of a single protein, or for the induction of more than one protein, which has application in the engineering and study of artificial networks. Importantly, conditional control of protein function is not exclusive of genetic manipulation. Ultimately therefore, as gene-editing ‘knockin’ strategies mature [131], it may become routine to incorporate any number of different conditional domains on a genome-wide scale to facilitate such studies.

## Abbreviations

AID: auxin-inducible degron
AP: adaptor protein
BD: binding domain
BET: bromo- and extra-terminal
CALI: chromophore-assisted light inactivation
CaMKIIα: Ca^2+^/calmodulin-dependent protein kinase IIα
CIB1: cryptochrome-interacting basic helix–loop–helix 1
CID: chemical inducers of dimerization
CLICR: clustering indirectly using cryptochrome 2
Cry: cryptochrome
DHFR: dihydrofolate reductase
ERRα: oestrogen-related receptor α
FALI: fluorophore-assisted light inactivation
FKBP: FK506-binding protein
FlAsH: fluorescein-based arsenical hairpin binder
FRAP: FKBP-rapamycin-associated protein
FRB: FKBP-rapamycin-binding
β-gal: β-galactosidase
Gal4/UAS: Gal4 upstream activating sequence
GPCR: G-protein-coupled receptor
HDR: homologous DNA recombination
IMiD: immunomodulatory drug
KS: knocksideways
LARIAT: light-activated reversible inhibition by assembled trap
LID: light-induced dimerization
LOV: light–oxygen–voltage
mTOR: mammalian target of rapamycin
MTX: methotrexate
PA-Rac: photoactivatable Race
PhyB: phytochrome B
PIF3: phytochrome-interacting factor 3
PROTAC: Proteolysis-Targeting Chimaera
ReAsH: resorufin-based arsenical hairpin binder
ROS: reactive oxygen species
RTK: receptor tyrosine kinase
Syt I: synaptotagmin I
TEV: tobacco etch virus
TIPI: TEV protease-mediated induction of protein instability
TIR1: transport inhibitor response 1
tsDHFR: temperature-sensitive dihydrofolate reductase
VHL: von Hippel-Lindau protein
